# A growth-based platform for detecting domain–peptide interactions in the cytoplasm of mammalian cells

**DOI:** 10.1038/s41598-022-22770-4

**Published:** 2022-10-27

**Authors:** Yosuke Kimura, Daiki Kashima, Masahiro Kawahara

**Affiliations:** 1 Department of Chemistry and Biotechnology, Graduate School of Engineering, The University of Tokyo, 7-3-1 Hongo, Bunkyo-Ku, Tokyo, 113-8656 Japan; 2 Laboratory of Cell Vaccine, Center for Vaccine and Adjuvant Research (CVAR), National Institutes of Biomedical Innovation, Health and Nutrition (NIBIOHN), 7-6-8 Saito-Asagi, Ibaraki-Shi, Osaka, 567-0085 Japan

**Keywords:** Biotechnology, Molecular engineering, Protein design, Synthetic biology, Genetic engineering

## Abstract

Development of a method for detecting protein–protein interactions (PPIs) in living cells is important for therapeutic drug screening against various diseases including infectious diseases. We have recently developed a method named SOS localization-based interaction screening (SOLIS), in which we designed membrane-anchored and SOS-fused chimeric proteins, whose PPI-dependent association triggers membrane localization of the SOS-fused chimeric protein, activates the Ras/MAPK pathway, and induces cell growth. While SOLIS was able to detect relatively strong PPIs, further sensitivity was required for detecting intracellular endogenous PPIs typically having a micromolar order of dissociation constant (*K*_d_). Here we develop high-sensitive SOLIS (H-SOLIS) that could universally detect PPIs with lower affinities. In order to improve the sensitivity, H-SOLIS introduces a heterodimeric helper interaction, in which addition of a small-molecule helper ligand could accommodate association of the two chimeric proteins and regulate the sensitivity. Four types of domain–peptide interactions having known *K*_d_ values are employed to examine the versatility and detection limit of H-SOLIS. Consequently, the heterodimer-inducible helper ligand dramatically enhances detection sensitivity, lowering the detection limit to a ten-micromolar order of *K*_d_. Thus, H-SOLIS could be a platform to detect disease-related domain–peptide interactions for drug discovery screening.

## Introduction

The coronavirus pandemic is shaking the world, generating many infected people, and afflicting humankind. To our surprise, mRNA vaccines have been developed so rapidly after discovery of the virus and immediately supplied worldwide^1,2^. However, it will be increasingly important to develop therapeutic agents to diminish the severity of infected patients^3^. Since the monkeypox virus and the highly pathogenic influenza virus might cause another pandemic in the future^4,5^, we face an urgent issue for speedy development of effective therapeutic agents to prepare for forthcoming infectious disease emergencies. Many intracellular proteins, including the pathogenic proteins of infectious diseases, exert their functions by interaction with other proteins. Therefore, development of a method for detecting protein–protein interactions (PPIs) in living cells is important for therapeutic drug screening against various diseases including infectious diseases. Conventionally, fluorescence or bioluminescence resonance energy transfer (FRET or BRET) systems and protein fragment complementation assays have been widely used to detect intracellular PPIs in living cells^6–8^. In these methods, genetically encoded chimeric proteins are designed, in which target and probe proteins are fused. Exogenous expression of the chimeric proteins in cells after gene transfer induces PPI-dependent readouts based on FRET, BRET, and enzyme activity. While these methods adopt transient expression systems enabling fast detection, off-target signals are relatively high because the chimeric proteins are overexpressed to improve the detection sensitivity^9–11^.

To ameliorate this issue, we previously developed a chimeric receptor-based system, in which target proteins are fused with the intracellular signaling domain of cytokine receptors. PPI between the target proteins triggers receptor dimerization and subsequent growth signaling, leading to cell growth as a readout. Since we employed c-Kit or thrombopoietin receptor as the intracellular signaling domain, we named c-Kit-based PPI screening (KIPPIS)^12–14^ or thrombopoietin receptor-based PPI screening (THROPPIS)^15^, respectively. KIPPIS and THROPPIS constitutively express chimeric receptors through stable retroviral transduction, which prevents off-target signals compared to the conventional PPI detection systems using transient overexpression. Furthermore, since PPI-dependent dimerization is prerequisite for growth signaling, false positives are less likely to occur. However, there is a common limitation of KIPPIS and THROPPIS; when a homodimeric protein is incorporated as a target protein, off-target growth signaling occurs irrespective of interaction with the other target protein. As a method to complement this limitation, we have recently developed a method named SOS localization-based interaction screening (SOLIS)^16^, in which we employed a SOS protein that activates the Ras/MAPK pathway and induces cell growth when localized at the plasma membrane. In SOLIS, a membrane localization sequence CaaX is fused at the C-terminus of a target protein, whereas the catalytic domain of SOS (SOS_cat_) is fused at the C-terminus of the other target protein. When the two chimeric proteins are co-expressed intracellularly and interact with each other through PPI between the target proteins, SOS_cat_ is membrane-localized, leading to cell growth signaling. In SOLIS, strictly IL-3-dependent Ba/F3 cells are employed to attain cell growth-based PPI detection in the IL-3-deprived condition. Because the growth signaling is solely dependent on the PPI-dependent localization of the SOS_cat_ chimera, SOLIS targets relatively strong interactions such as antigen–antibody interactions with a nanomolar order of dissociation constant (*K*_d_). In fact, relatively strong interactions between MDM2–p53 peptide in a thioredoxin scaffold (*K*_d_ = 140 nM) and GFP–anti-GFP nanobody (*K*_d_ = 0.32–33 nM) were successfully detected in SOLIS^16^.

To date, many small-molecule and peptide inhibitors targeting intracellular domain–peptide interactions have been reported^17–19^. Such domain–peptide interactions typically have a micromolar order of *K*_d_, which necessitates improving sensitivity of SOLIS. Here we aim to develop high-sensitive SOLIS (H-SOLIS) that could universally detect PPIs with lower affinities. In order to improve the sensitivity, H-SOLIS introduces a heterodimeric helper interaction, in which addition of a small-molecule helper ligand could accommodate association of the two chimeric proteins and regulate the sensitivity. Four types of domain–peptide interactions having known *K*_d_ values are employed to examine the versatility and detection limit of H-SOLIS.

## Results

### Designing chimeric proteins in H-SOLIS

**Figure 1:**
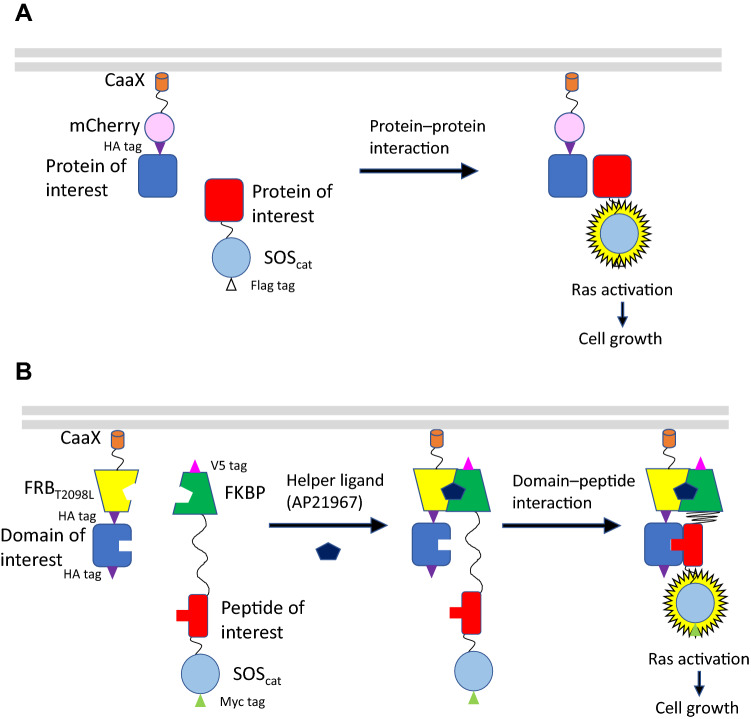
The design and activation mechanism of original SOLIS and H-SOLIS. (**A**) Schematic illustration of original SOLIS. (**B**) Schematic illustration of H-SOLIS.

We firstly designed two chimeric proteins for H-SOLIS to attain improved sensitivity compared to the previously developed original SOLIS system (Fig. 1). One is a membrane-anchored chimera composed of a domain of interest, a T2098L mutant of the FRB domain of FKBP12-rapamycin associated protein (FRB_T2098L_), and a CaaX motif for membrane anchoring through lipid modification at the C-terminus. The other is a signaling chimera composed of FK506-binding protein 12 (FKBP), a peptide of interest, and the catalytic domain of SOS (SOS_cat_). The association between the domain and peptide of interest induces membrane localization of the signaling chimera, which activates membrane-localized endogenous Ras protein through the GDP/GTP exchanging activity of SOS_cat_, leading to growth signaling via the Ras/MAPK pathway. A small-molecule heterodimeric ligand AP21967 induces heterodimerization between FRB_T2098L_ and FKBP^20^, which facilitates interaction between the domain and peptide of interest. Thus, even weak association between the domain and peptide of interest could be captured owing to the helper interaction between FRB_T2098L_ and FKBP. To minimize off-target growth signaling with only the helper interaction, a relatively long flexible linker (G_4_S)_5_ is inserted between FKBP and the peptide of interest in the signaling chimera.

### Proof of concept for H-SOLIS using MDM2–p53 interaction

**Figure 2:**
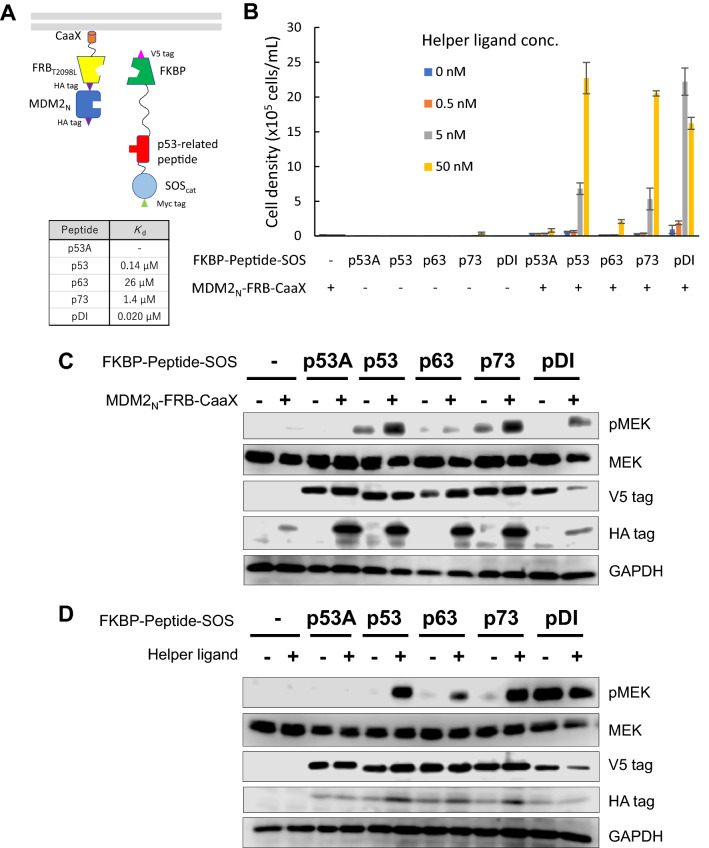
Proof of concept for H-SOLIS using MDM2–p53 interaction. (**A**) The design of the membrane-anchored and signaling chimeras incorporating MDM2_N_ and the p53-related peptides. The dissociation constants of each peptide binding to MDM2_N_ are also shown. (**B**) Cell growth assay. Cells were seeded at 1 × 10^5^ cells/mL and cultured for 3 days in the medium with the indicated concentrations of the helper ligand AP21967. Viable cell densities are shown as mean ± SD (n = 3, biological replicates). (**C**) Comparison of signaling properties of the cells with or without MDM2_N_-FRB-CaaX expression. Cells were stimulated for 15 min with 50 nM of the helper ligand AP21967 for all samples commonly. Phospho-MEK, whole MEK, V5 tag (FKBP-Peptide-SOS expression), HA tag (MDM2_N_-FRB-CaaX expression), and GAPDH (loading control) were detected using corresponding primary antibodies. (**D**) The helper ligand-dependent signaling of the cells commonly expressing MDM2_N_-FRB-CaaX. Cells were stimulated for 15 min with or without 50 nM of the helper ligand AP21967. Uncropped blot images for (**C**) and (**D**) are provided in Supplementary Fig. 3.

To prove the concept for H-SOLIS, we firstly chose the N-terminal domain of murine double minute 2 (MDM2_N_) as a domain of interest, because there are several interacting partner peptides with a range of dissociation constants (*K*_d_) (Fig. 2A). p53 is an endogenous MDM2_N_-interacting partner and the peptide at the interaction interface was chosen (*K*_d_ = 0.14 μM)^21^. A three-amino-acid mutant of the p53 peptide critical for binding with MDM2_N_ was used as a negative control (p53A)^12,22^. As p53-related peptides, pDI (*K*_d_ = 0.020 μM), p73 (*K*_d_ = 1.4 μM), and p63 (*K*_d_ = 26 μM) peptides were also employed to examine the detection limit of H-SOLIS^21,23^.

Retroviral vectors encoding the membrane-anchored and signaling chimeras were constructed, in which puromycin and blasticidin resistance genes were encoded respectively. IL-3-dependent Ba/F3 cells were employed as host cells also in H-SOLIS to detect domain–peptide interactions with cell growth in the absence of IL-3. Ba/F3 cells were transduced with the retroviral vectors, followed by antibiotics selection to establish single- and co-transduced cells. To investigate growth properties of the transduced cells, we performed a growth assay, in which the cells were cultured with various concentrations of the helper ligand AP21967 (Fig. 2B). Consequently, the co-transduced cells with the pDI peptide most vigorously grew in a helper ligand-dependent manner. The co-transduced cells with the lower-affinity peptides (p53 and p73) grew moderately. Intriguingly, the co-transduced cells with the lowest affinity peptide (p63) also grew in the presence of 50 nM of the helper ligand. This growth, albeit to a lesser extent, was significant because the co-transduced cells with the negative control peptide (p53A) did not grow under the same condition. In addition, all single-transduced cells as negative controls did not grow in all helper ligand concentrations. The results indicated that the heterodimeric helper interaction greatly improved the sensitivity of H-SOLIS, which enabled detection of the weak interaction (*K*_d_ = 26 μM).

To visualize differential MEK phosphorylation events downstream of the activation of the signaling chimeras, the transduced cells maintained with IL-3 were cultured without IL-3 for 6 h, stimulated with the helper ligand for 15 min, lysed, and subjected to Western blot analysis. As a result, the co-transduced cells with the interacting peptides (p53, p63, p73, and pDI) showed stronger phosphorylation of MEK than the single-transduced cells (Fig. 2C). When the co-transduced cells were compared between non-stimulated and helper ligand-stimulated conditions, phosphorylation of MEK was significantly induced by the helper stimulation in the p53, p63, and p73 peptides, while the pDI peptide induced sufficient phosphorylation of MEK even in the non-stimulated condition (Fig. 2D). In both experiments, the no-chimera control (-) and negative control peptide (p53A) did not induce phosphorylation of MEK. These results clearly proved the concept of H-SOLIS and demonstrated high-sensitive detection of the MDM2_N_–p53-related peptide interactions.

### Demonstrating versatility of H-SOLIS

**Figure 3:**
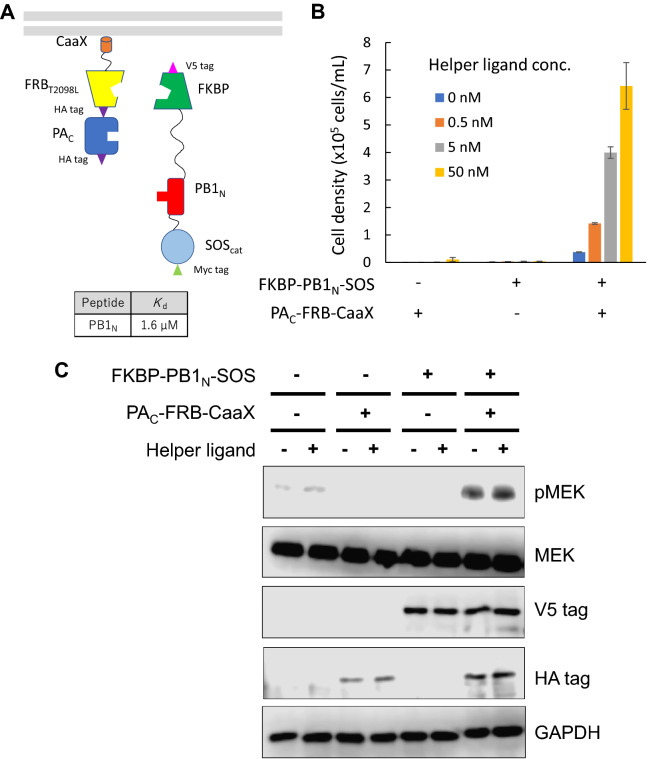
Detecting PA_C_–PB1_N_ interaction by H-SOLIS. (**A**) The design of the membrane-anchored and signaling chimeras incorporating PA_C_ and PB1_N_. The dissociation constant of PB1_N_ binding to PA_C_ is also shown. (**B**) Cell growth assay. Cells were seeded at 1 × 10^5^ cells/mL and cultured for 3 days in the medium with the indicated concentrations of the helper ligand AP21967. Viable cell densities are shown as mean ± SD (n = 3, biological replicates). (**C**) Signaling analysis. Cells were stimulated for 15 min with or without 50 nM of the helper ligand AP21967. Phospho-MEK, whole MEK, V5 tag (FKBP-PB1_N_-SOS expression), HA tag (PA_C_-FRB-CaaX expression), and GAPDH (loading control) were detected using corresponding primary antibodies. Uncropped blot images are provided in Supplementary Fig. 3.

To examine versatility of H-SOLIS, we chose the C-terminal domain of polymerase acidic protein (PA_C_) of the influenza virus as a domain of interest (Fig. 3A). PA_C_ interacts with the N-terminal peptide of polymerase basic protein 1 (PB1_N_) of the influenza virus (*K*_d_ = 1.6 μM)^24^. Ba/F3 cells were transduced with retroviral vectors encoding the PA_C_-fused membrane-anchored chimera and PB1_N_-fused signaling chimera, followed by antibiotics selection to establish single- and co-transduced cells. A growth assay clearly revealed helper ligand dose-dependent growth of the co-transduced cells, demonstrating a dramatic increase of detection sensitivity through the helper interaction (Fig. 3B). Western blot analysis showed phosphorylation of MEK in the co-transduced cells (Fig. 3C). However, the phosphorylation levels were not significantly induced by the helper ligand. Again, the single-transduced cells as negative controls were non-responsive to the helper ligand, inducing neither growth nor phosphorylation of MEK. These results indicate that H-SOLIS can be used for detecting inter-subunit interactions derived from viral proteins in the cytoplasm of mammalian cells.

**Figure 4:**
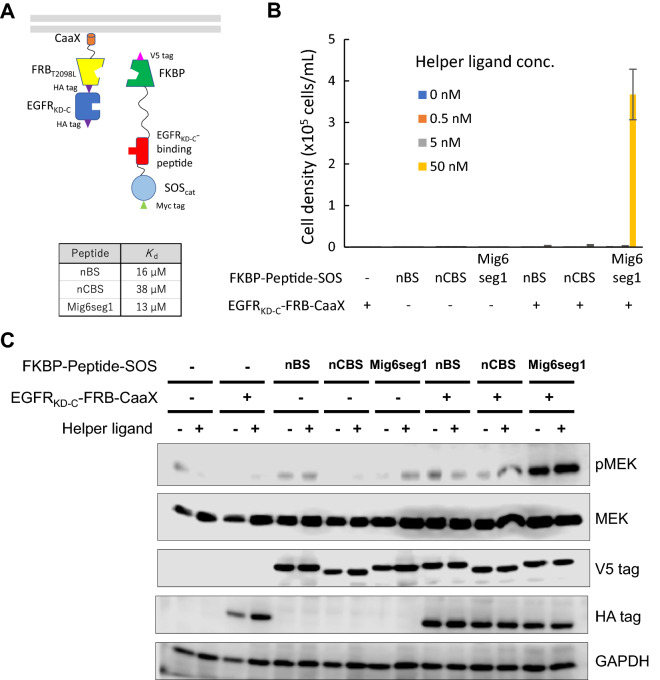
Detecting interactions between EGFR_KD-C_ and its binding peptides by H-SOLIS. (**A**) The design of the membrane-anchored and signaling chimeras incorporating EGFR_KD-C_ and its binding peptides. The dissociation constants of each peptide binding to EGFR_KD-C_ are also shown. (**B**) Cell growth assay. Cells were seeded at 1 × 10^5^ cells/mL and cultured for 3 days in the medium with the indicated concentrations of the helper ligand AP21967. Viable cell densities are shown as mean ± SD (n = 3, biological replicates). (**C**) Signaling analysis. Cells were stimulated for 15 min with or without 50 nM of the helper ligand AP21967. Phospho-MEK, whole MEK, V5 tag (FKBP-Peptide-SOS expression), HA tag (EGFR_KD-C_-FRB-CaaX expression), and GAPDH (loading control) were detected using corresponding primary antibodies. Uncropped blot images are provided in Supplementary Fig. 3.

To further demonstrate utility of H-SOLIS, we next focused on weak interactions between endogenous proteins. The kinase domain of epidermal growth factor receptor (EGFR) is composed of the N-lobe and C-lobe, which form asymmetric homodimers between two receptor chains. The C-lobe of one receptor chain (activator) docks with the N-lobe of the other (receiver), which triggers activation of the kinase domain^25^. A recent study identified two N-lobe-derived peptides named N-terminal binding sequence (nBS) and N-terminal core binding sequence (nCBS) that could bind to the C-lobe^26^. Furthermore, a peptide segment derived from mitogen-inducible gene 6 (Mig6seg1) binds to the C-lobe and inhibits dimerization of EGFR^27^. With these facts in mind, we chose the C-lobe of the kinase domain of EGFR (EGFR_KD-C_) as a domain of interest to investigate whether relatively weak binding of nBS (*K*_d_ = 16 μM), nCBS (*K*_d_ = 38 μM), and Mig6seg1 (*K*_d_ = 13 μM) as peptides of interest could be detected in H-SOLIS (Fig. 4A). Ba/F3 cells were transduced with retroviral vectors encoding the EGFR_KD-C_-fused membrane-anchored chimera and nBS-, nCBS- or Mig6seg1-fused signaling chimera, followed by antibiotics selection to establish single- and co-transduced cells. Consequently, only the co-transduced cells with Mig6seg1 grew in the highest dose (50 nM) of the helper ligand (Fig. 4B). MEK was strongly phosphorylated in the co-transduced cells with Mig6seg1 without helper ligand dependency (Fig. 4C). The single-transduced cells and the other co-transduced cells induced weaker phosphorylation of MEK. These results indicate that H-SOLIS was able to detect interaction of Mig6seg1 with EGFR_KD-C_, but failed to detect interaction of nBS and nCBS with EGFR_KD-C_.

**Figure 5:**
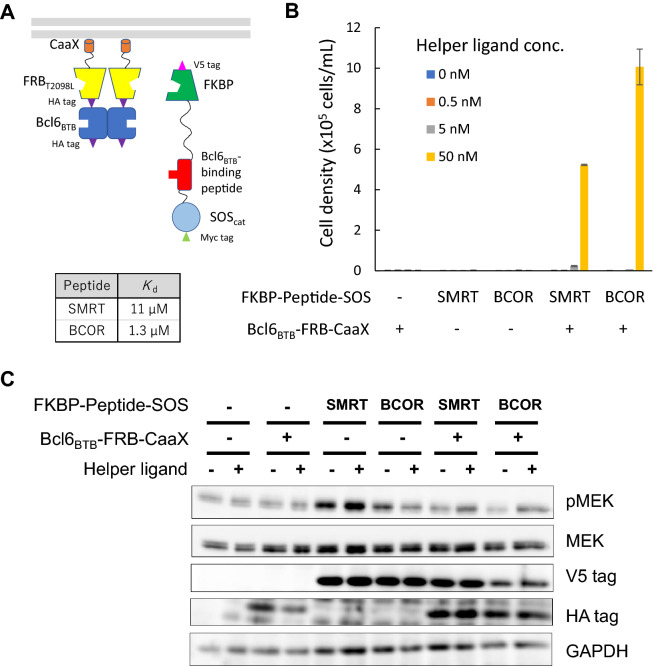
Detecting interactions between Bcl6_BTB_ and its binding peptides by H-SOLIS. (**A**) The design of the membrane-anchored and signaling chimeras incorporating Bcl6_BTB_ and its binding peptides. The dissociation constants of each peptide binding to Bcl6_BTB_ are also shown. (**B**) Cell growth assay. Cells were seeded at 1 × 10^5^ cells/mL and cultured for 3 days in the medium with the indicated concentrations of the helper ligand AP21967. Viable cell densities are shown as mean ± SD (n = 3, biological replicates). (**C**) Signaling analysis. Cells were stimulated for 15 min with or without 50 nM of the helper ligand AP21967. Phospho-MEK, whole MEK, V5 tag (FKBP-Peptide-SOS expression), HA tag (Bcl6_BTB_-FRB-CaaX expression), and GAPDH (loading control) were detected using corresponding primary antibodies. Uncropped blot images are provided in Supplementary Fig. 3.

An advantage of H-SOLIS compared to our previously developed KIPPIS and THROPPIS systems is that H-SOLIS could be applied to homo-oligomeric proteins in principle. To demonstrate this aspect, we chose the BTB domain of B-cell lymphoma 6 (Bcl6_BTB_) as a homodimeric domain of interest (Fig. 5A). Bcl6 is a transcriptional repressor, which plays an important role in germinal center formation and antibody affinity maturation in B cells^28^. Bcl6 forms homodimers via the BTB domain, to which co-repressors bind and execute transcriptional repression. We chose two peptides derived from the corepressors SMRT (*K*_d_ = 11 μM) and BCOR (*K*_d_ = 1.3 μM) as peptides of interest^29,30^. Single- and co-transduced cells were established with retroviral vectors encoding the Bcl6_BTB_-fused membrane-anchored chimera and SMRT- or BCOR-fused signaling chimera. Intriguingly, the co-transduced cells with SMRT or BCOR grew in the highest dose (50 nM) of the helper ligand, while the single-transduced and non-transduced cells did not grow (Fig. 5B). Signaling analysis showed unexpected phosphorylation levels of MEK (Fig. 5C). The single transduced cells with SMRT exhibited maximal phosphorylation levels irrespective of stimulation with the helper ligand. Moreover, the co-transduced cells with SMRT or BCOR showed relatively weak but helper ligand-induced phosphorylation of MEK. Taken together, the results demonstrated that H-SOLIS accommodated the homodimeric domain of interest and the detection limit was estimated as a ten-micromolar order of *K*_d_.

## Discussion

In this study, we designed H-SOLIS as a high-sensitive platform to detect cytoplasmic PPIs in IL-3-dependent Ba/F3 cells. The chimeric proteins designed were the membrane-anchored and signaling chimeras, whose association was regulated by the heterodimeric helper ligand as well as the domain and peptide of interest. Thus, membrane localization of the signaling chimera was greatly assisted by the helper ligand even for weak domain–peptide interactions. We employed an MDM2_N_–p53 system to prove the concept of H-SOLIS using p53-related peptides with a range of affinities toward MDM2_N_. Intriguingly, the weak MDM2_N_–p63 peptide interaction (*K*_d_ = 26 μM) was successfully detected in the presence of the helper ligand. Interestingly, the H-SOLIS assay was able to detect the domain–peptide interactions without a thioredoxin scaffold, which has been utilized for peptide presentation in the original SOLIS. Thus, H-SOLIS attained a dramatic increase of sensitivity and stability by introducing the helper interaction.

To investigate robustness and versatility of H-SOLIS, we tested other domain–peptide interactions. The PA_C_–PB1_N_ (*K*_d_ = 1.6 μM), EGFR_KD-C_–Mig6seg1 (*K*_d_ = 13 μM), Bcl6_BTB_–SMRT (*K*_d_ = 11 μM), and Bcl6_BTB_–BCOR (*K*_d_ = 1.3 μM) interactions were successfully detected, which further demonstrated that H-SOLIS is a versatile platform for detecting domain–peptide interactions with micromolar orders of *K*_d_. Furthermore, the interactions between the homodimeric domain Bcl6_BTB_ and its binding peptides were successfully detected. This aspect is advantageous over our previously developed KIPPIS and THROPPIS systems, in which homodimeric domains or peptides cannot be applied due to the homodimer-induced activation mechanism of c-Kit and thrombopoietin receptor.

Unexpectedly, “no ligand” conditions often led to sufficient MEK phosphorylation in spite of no cell growth, indicating that the MEK phosphorylation levels did not necessarily correlate with the cell growth levels. MEK phosphorylation, which was a short-term (15 min) event upon ligand stimulation, may not have matched with long-term (3 days) cell growth. Cell growth may require sustained levels of MEK phosphorylation, which would be difficult to demonstrate in the short-term stimulation experiments. For deeper understanding of this issue, we need to assess global phosphorylation levels of signaling molecules in a high-throughput way. In addition, the EGFR_KD-C_–nBS (*K*_d_ = 16 μM) and EGFR_KD-C_–nCBS (*K*_d_ = 38 μM) interactions failed to be detected. These *K*_d_ values were determined by measuring the association between the recombinant protein and synthetic peptides using fluorescence anisotropy in vitro^26^, which may not reflect the binding properties in the cytoplasm of mammalian cells. Since the EGFR_KD-C_–Mig6seg1 (*K*_d_ = 13 μM) interaction, which had been confirmed in mammalian cells in the previous literature^27^, was able to be detected, H-SOLIS may be useful as a tool for reliable detection of specific binders in the cytosol of living cells.

In H-SOLIS, a domain of interest needs to be fused with FRB_T2098L_ at the C-terminus, whereas a peptide of interest should be flanked by FKBP and SOS_cat_ at the N- and C-termini, respectively. This configuration may limit the applicability of H-SOLIS for polypeptides that require free extremity to interact with their partner. In addition, the N- to C-terminal order of different polypeptides of interest that constitute the membrane-anchored and signaling chimeras may affect outcome of the H-SOLIS assay, although the signaling chimera would be already flexible enough to accommodate steric effects. To ameliorate this issue, the membrane-anchored chimera could become more flexible by incorporating a flexible linker between FRB_T2098L_ and a domain of interest, which could further diminish steric effects in H-SOLIS. Nevertheless, H-SOLIS would be advantageous over other cell-based PPI detection systems because false negatives are less likely to occur owing to the conformation-insensitive nature of SOS_cat_ activation properties as revealed in the original SOLIS. Furthermore, the use of cytokine-dependent cells attains high signal-to-noise ratios of the entire system, which could allow cell growth-based positive selection of interacting peptides from a library.

In summary, H-SOLIS is useful as a system for detecting domain–peptide interactions in the cytoplasm of mammalian cells. The heterodimer-inducible helper ligand dramatically enhances detection sensitivity, lowering the detection limit to a ten-micromolar order of *K*_d_. Although we have focused on domain–peptide interactions in this study, H-SOLIS may also be applied to detect domain–domain and protein–protein interactions unless the membrane-anchored and signaling chimeras are sterically difficult to interact with each other for sufficient activation of SOS_cat_-mediated growth signaling. Thus, H-SOLIS could be a platform to detect disease-related polypeptide interactions for drug discovery screening.

## Materials and methods

### Plasmid construction

Retroviral backbone plasmids pMK-stuffer-IRES-Puro^R^ and pMK-stuffer- IRES-Blast^R^ were used for constructing plasmids encoding membrane-anchored and signaling chimeras, respectively^31,32^. The internal ribosomal entry site (IRES) facilitates bicistronic expression of the chimera gene and puromycin (Puro^R^) or blasticidin (Blast^R^) resistance gene in the backbone plasmids. To construct the plasmids encoding the chimeras, PCR-amplified host and insert sequences were fused by In-Fusion HD Cloning Kit (Takara Bio, Shiga, Japan), followed by standard plasmid subcloning procedures using an *E. coli* strain NEB Turbo (New England Biolabs, Ipswich, MA). The extracted plasmids were sequenced to identify subclones with correct sequences. The amino acid sequences of the membrane-anchored and signaling chimeras are summarized in Supplementary Information (Supplementary Figs. 1 and 2).

### Cell culture

For retroviral packaging, Plat-E cells (kindly provided by Dr. T. Kitamura, The University of Tokyo) were cultured in Dulbecco’s modified Eagle’s medium (DMEM) (Nissui Pharmaceutical, Tokyo, Japan) supplemented with 10% fetal bovine serum (Sigma-Aldrich, St. Louis, MO), 1 µg/ml puromycin (Sigma-Aldrich), and 10 µg/ml blasticidin (Kaken Pharmaceutical, Tokyo, Japan). Murine IL-3-dependent pro-B Ba/F3 cells (RIKEN Cell Bank #RCB0805, Ibaraki, Japan) were cultured in RPMI1640 medium (Nissui Pharmaceutical) supplemented with 10% fetal bovine serum and 1 ng/ml murine IL-3 (Kaico, Fukuoka, Japan).

### Retroviral transduction

Plat-E cells were seeded in 6-well plates a day before transfection. The cells were transfected with the retroviral plasmids using Lipofectamine LTX (Thermo Fisher Scientific, Waltham, MA) as described previously^15^. Two days later, each well of a 24-well plate was coated with 25 μg/ml Retronectin (Takara Bio, Shiga, Japan), blocked with 2% BSA/PBS, and washed with PBS at room temperature. The retroviral supernatant filtrated with Millex 13 mm Durapore PVDF 0.45 (Merck Millipore, Burlington, MA) was loaded into the Retronectin-coated 24-well plate to adsorb retroviral vectors on the wells by incubating for 5 h in a 37℃/5% CO_2_ incubator. After removing the retroviral supernatant, Ba/F3 cells (1 × 10^5^ cells/well) were added on the retrovirus-adsorbed wells for transduction. Two days later, the cells were trypsinized and seeded into a 24-well plate for drug resistance selection. The drug concentrations used are 2 µg/ml puromycin and 40 μg/ml blasticidin.

### Growth assay

Cells were washed twice with PBS and seeded at 1.0 × 10^5^ cells/ml into 24-well plates with or without various concentrations of AP21967 (Takara Bio). Three days later, cells were mixed with Flow-Count Fluorospheres (Beckman Coulter, Fullerton, CA) and 1 μg/ml propidium iodide (Sigma-Aldrich) and subjected to flow cytometry (FACSCalibur; BD Biosciences, Lexington, KY) to measure viable cell density. The data were analyzed using FlowJo software (BD Biosciences).

### Signaling analysis

Cells were washed twice with PBS and cultured in the medium devoid of IL-3 for 6 h. The cells (1.0 × 10^6^ cells) were collected by centrifugation and resuspended with 500 μl of the culture medium with or without 50 nM AP21967 for 15 min at 37 ℃. The cells were washed twice with ice-cold PBS containing 1 mM Na_3_VO_4_. The cells were solubilized with 100 μl of lysis buffer (20 mM HEPES (pH 7.5), 150 mM NaCl, 10% glycerol, 1% Triton X-100, 1.5 mM MgCl_2_, 1 mM EGTA, 10 μg/ml aprotinin, 10 μg/ml leupeptin, 1 mM Na_3_VO_4_), incubated for 10 min on ice, and centrifuged at 16,100 g for 10 min at 4 ℃. The supernatant was mixed with 33 μl of 4 × Laemmli’s sample buffer and boiled at 98 ℃ for 5 min. The cell lysates were resolved by SDS-PAGE and transferred on a nitrocellulose membrane (Cytiva, Tokyo, Japan). After blocking with 3% BSA (Sigma-Aldrich), the membrane was incubated with rabbit primary antibodies and subsequently with horseradish peroxidase-conjugated anti-rabbit IgG (Thermo Fisher Scientific). The blots were visualized by adding Luminata Forte Western HRP substrate (Merck Millipore) on the membrane. The chemiluminescence readout was measured on a C-DiGit scanner (LI-COR Biosciences, Lincoln, NE). The rabbit primary antibodies used are anti phospho-MEK1/2 (S217/221)(Cell Signaling Technology, Danvers, MA), anti MEK1/2 (Cell Signaling Technology), anti V5 tag (Merck Millipore), anti HA tag (Bethyl Laboratories, Montgomery, TX), and anti GAPDH (Santa Cruz Biotechnology, Santa Cruz, CA).

## Supplementary Information

Supplementary Information.

## Data Availability

All data generated or analysed during this study are included in this published article and its Supplementary Information.
