# Supplementary material for: A growth-based platform for detecting domain–peptide interactions in the cytoplasm of mammalian cells
Source: Sci Rep. 2022 Oct 27;12:18028. doi: 10.1038/s41598-022-22770-4 (PMC9607845; doi:10.1038/s41598-022-22770-4)
Supplement: Supplementary file 1 — Supplementary Information. [file 41598_2022_22770_MOESM1_ESM.pdf]

Supplementary Information

A growth-based platform for detecting domain–peptide interactions in the cytoplasm of mammalian cells

Yosuke Kimura[1], Daiki Kashima[1], Masahiro Kawahara[1,2,*]

[1]Department of Chemistry and Biotechnology, Graduate School of Engineering, The University of Tokyo, 7-3-1 Hongo, Bunkyo-ku, Tokyo 113-8656, Japan

[2]Laboratory of Cell Vaccine, Center for Vaccine and Adjuvant Research (CVAR), National Institutes of Biomedical Innovation, Health and Nutrition (NIBIOHN), 7-6-8 Saito-Asagi, Ibaraki-shi, Osaka 567-0085, Japan

[*]Correspondence: Masahiro Kawahara, Laboratory of Cell Vaccine, Center for Vaccine and Adjuvant Research (CVAR), National Institutes of Biomedical Innovation, Health and Nutrition (NIBIOHN), 7-6-8 Saito-Asagi, Ibaraki-shi, Osaka 567-0085, Japan.
E-mail: m-kawahara@nibiohn.go.jp

MYPYDVPDYAdomainYPYDVPDYTRILWHEMWHEGLEEASRLYFGERNVKGMFEVL
EPLHAMMERGPQTLKETSFNQAYGRDLMEAQEWCRKYMKSGNVKDLLQAWDLYY
HVFRRISKAAAGGGGSGGGGSGGGGSKKKKKKSKTKCVIM

$MDM2_N$

ASEQETLVRPKPLLLKLLKSVGAQKDTYTMKEVLFYLGQYIMTKRLYDEKQQHIVYC
SNDLLGDLFGVPSFSVKEHRKIYTMIYRNLVVVNQQ

PA_C

MNGYIEGKLSQMSKEVNARIEPFLKTTPRPLRLPNGPPCSQRSKFLLMDALKLSIEDPS
HEGEGIPLYDAIKCMRTFFGWKEPNVVKPHEKGINPNYLLSWKQVLAELQDIENEEKI
PKTKNMKKTSQLKWALGENMAPEKVDFDDCKDVGDLKQYDSDEPELRSLASWIQN
EFNKACELTDSSWIELDEIGEDVAPIEHIASMRRNYFTSEVSHCRATEYIMKGVYINTA
LLNASCAAMDDFQLIPMISKCRTKEGRRKTNLYGFIIKGRSHLRNDTDVVNFVSMEFS
LTDPRLEPHKWEKYCVLEIGDMLIRSAIGQVSRPMFLYVRTNGTSKIKMKWGMEMRR
CLLQSLQQIESMIEAESSVKEKDMTKEFFENKSETWPIGESPKGVEESSIGKVCRTLLA
KSVFNSLYASPQLEGFSAESRKLLLIVQALRDNLEPGTFDLGGLYEAIEECLINDPWVL
LNASWFNSFLTHALS

$EGFR_{KD\text{-}C}$

ALLRQLKETEFKKIKVLGSGAFGTVYKGLWIPEGEKVKIPVAIKELREATSPKANKEIL
DEAYVMASVDNPHVCRLLGICLTSTVQLITQLMPFGCLLDYVREHKDNIGSQYLLNW
CVQIAKGMNYLEDRRLVHRDLAARNVLVKTPQHVKITDFGLAKLLGAEEKEYHAEG
GKVPIKWMALESILHRIYTHQSDVWSYGVTVWELMTFGSKPYDGIPASEISSILEKGE
RLPQPPICTIDVYMIMVKCWMIDADSRPKFRELIIEFSKMARDPQRYLVIQGDERMHL
PSPTDSNFYRALMDEEDMDDVVDADEYLIPQQG

$Bcl6_{BTB}$

ADSCIQFTRHASDVLLNLNRLRSRDILTDVVIVVSREQFRAHKTVLMACSGLFYSIFTD
QLKCNLSVINLDPEINPEGFCILLDFMYTSRLNLREGNIMAVMATAMYLQMEHVVDT
CRKFIKASE

Supplementary Figure 1 The amino acid sequences of membrane-anchored chimeras. Black: linker or extra sequence. Purple: HA tag. Yellow: FRB_{T2098L}. Orange: CaaX sequence.

MGKPIPNPLLGLDSTGSGGVQVETISPGDGRTFPKRGQTCVVHYTGMLEDGKKFDSS
RDRNKPFKFMLGKQEVIRGWEEGVAQMSVGQRAKLTISPDYAYGATGHPGIIPPHATL
VFDVELLKAAAGGGGSGGGGSGGGGSGGGGSGGGGSQLpeptideGSSGGGGSGGGGS
TQMRLPSAEVYRFAEPDSEENILFEENVQPKAGIPIIKAGTVLKLIERLTYHMYADPNF
VRTFLTTYRSFCRPQELLSLLIERFEIPEPEPTEADRIAIENGDQPLSAELKRFRKEYIQP
VQLRVLNVCRHWVEHHFYDFERDADLLQRMEEFIGTVRGKAMKKWVESITKIIQRK
KIARDNGPGHNITFQSSPPTVEWHISRPGHIETFDLLTLHPIEIARQLTLLESDLYRAVQP
SELVGSVWTKEDKEINSPNLLKMIRHTTNLTLWFEKCIVETENLEERVAVVSRIIEILQV
FQELNNFNGVLEVVSAMNSSPVYRLDHTFEQIPSRQKKILEEAHELSEDHYKKYLAK
LRSINPPCVPFFGIYLTNILKTEEGNPEVLRRHGKELINFSKRRRVAEITGEIQQYQNQP
YCLRVEPDIKRFFENLNPMGNSMEKEFTDYLFNKSLEIEPRHPKPLPRFPKKYSYPLKS
PGVRPSNPRPGTGGIEQKLISEEDL

p53A SQETASDLAKLAPEN
p53 SQETFSDLWKLLPEN
p63 SPEVFQHIWDFLEQP
p73 GGTTFEHLWSSLEPD
pDI LTFEHYWAQLTS

PB1$_N$ MDVNPTLLFLKVPAQ

nBS LVEPLTPSAEAPNQALLRILKET
nCBS NQALLRILKE
Mig6seg1 SPKSLPSYLNGVMPPTQSFAPDPKYVSSKA

SMRT LVATVKEAGRSIHEIPR
BCOR RSEIISTAPSSWVVPGP

Supplementary Figure 2 The amino acid sequences of signaling chimeras.
Black: linker or extra sequence. Pink: V5 tag. Green: FKBP. Light blue: SOS_{cat}. Light green: Myc tag.

Fig. 2C

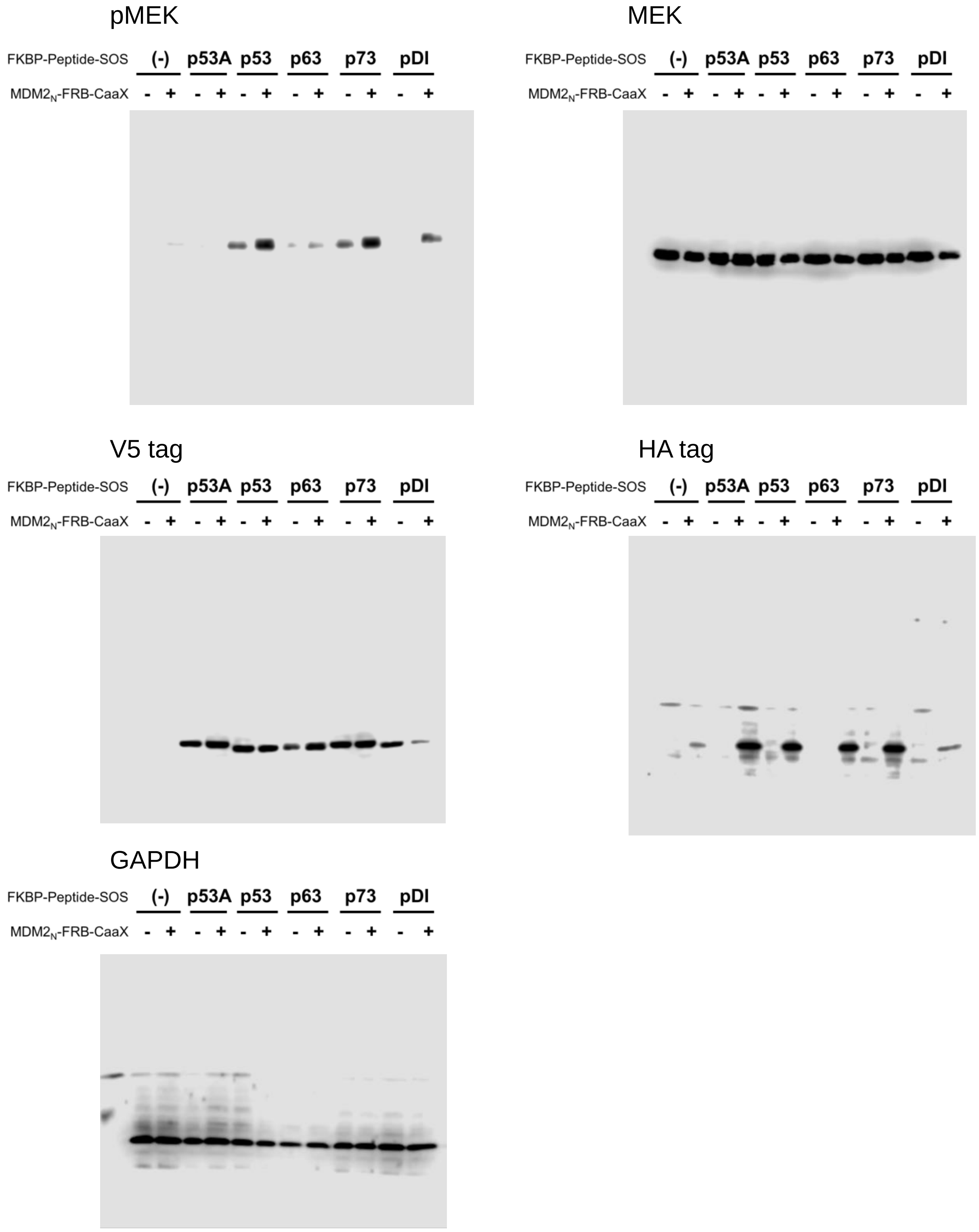
pMEK
FKBP-Peptide-SOS (-) p53A p53 p63 p73 pDI
MDM2$_N$-FRB-CaaX - + - + - + - + - + - +
MEK
FKBP-Peptide-SOS (-) p53A p53 p63 p73 pDI
MDM2$_N$-FRB-CaaX - + - + - + - + - + - +
V5 tag
FKBP-Peptide-SOS (-) p53A p53 p63 p73 pDI
MDM2$_N$-FRB-CaaX - + - + - + - + - + - +
HA tag
FKBP-Peptide-SOS (-) p53A p53 p63 p73 pDI
MDM2$_N$-FRB-CaaX - + - + - + - + - + - +
GAPDH
FKBP-Peptide-SOS (-) p53A p53 p63 p73 pDI
MDM2$_N$-FRB-CaaX - + - + - + - + - + - +

Fig. 2D

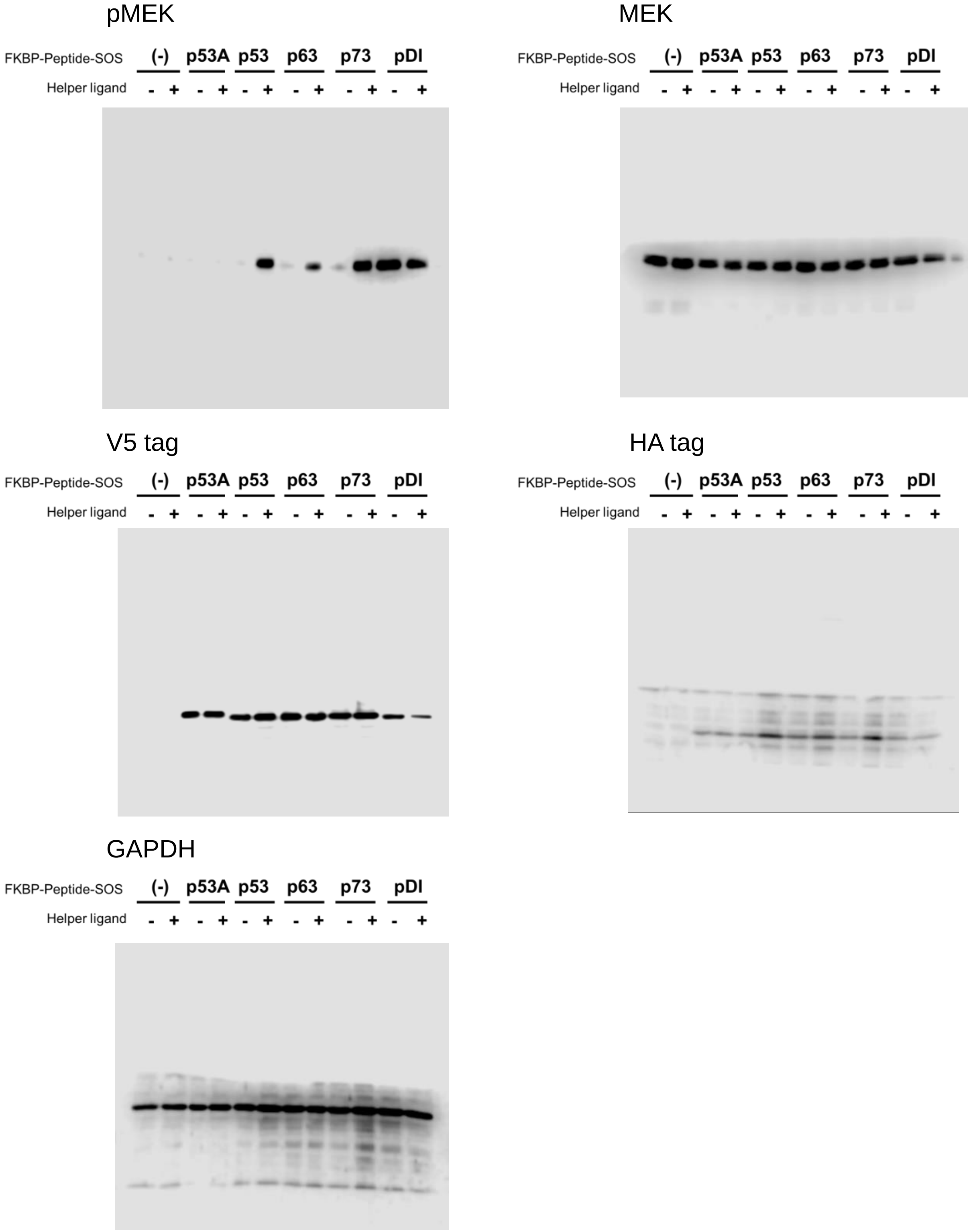
pMEK
MEK
V5 tag
HA tag
GAPDH
FKBP-Peptide-SOS
(-)
p53A
p53
p63
p73
pDI
Helper ligand
- + - + - + - + - + - +

Fig. 3C

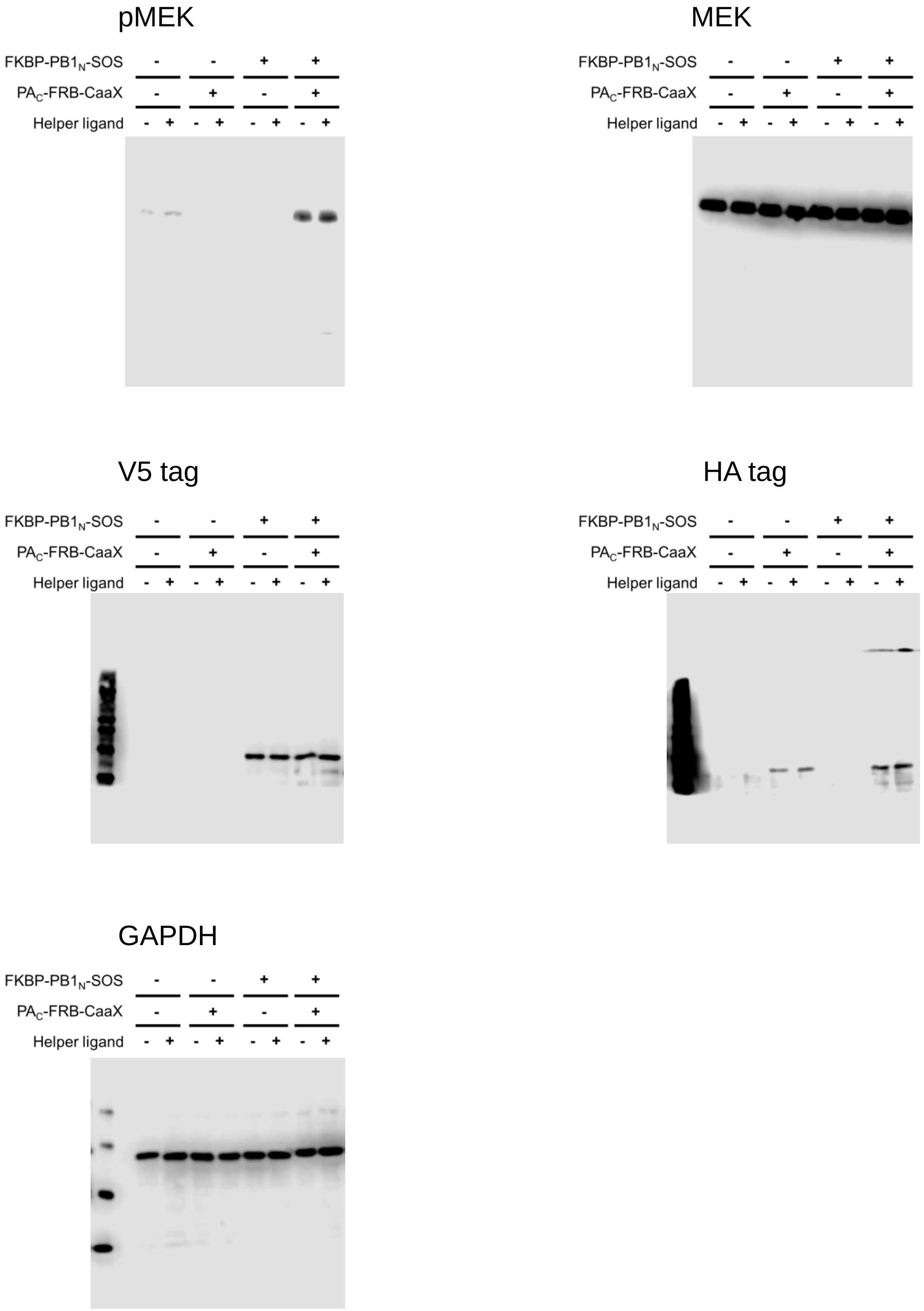
pMEK
FKBP-PB1$_N$-SOS - - + +
PA$_C$-FRB-CaaX - + - +
Helper ligand - + - + - + - +
MEK
FKBP-PB1$_N$-SOS - - + +
PA$_C$-FRB-CaaX - + - +
Helper ligand - + - + - + - +
V5 tag
FKBP-PB1$_N$-SOS - - + +
PA$_C$-FRB-CaaX - + - +
Helper ligand - + - + - + - +
HA tag
FKBP-PB1$_N$-SOS - - + +
PA$_C$-FRB-CaaX - + - +
Helper ligand - + - + - + - +
GAPDH
FKBP-PB1$_N$-SOS - - + +
PA$_C$-FRB-CaaX - + - +
Helper ligand - + - + - + - +

Fig. 4C

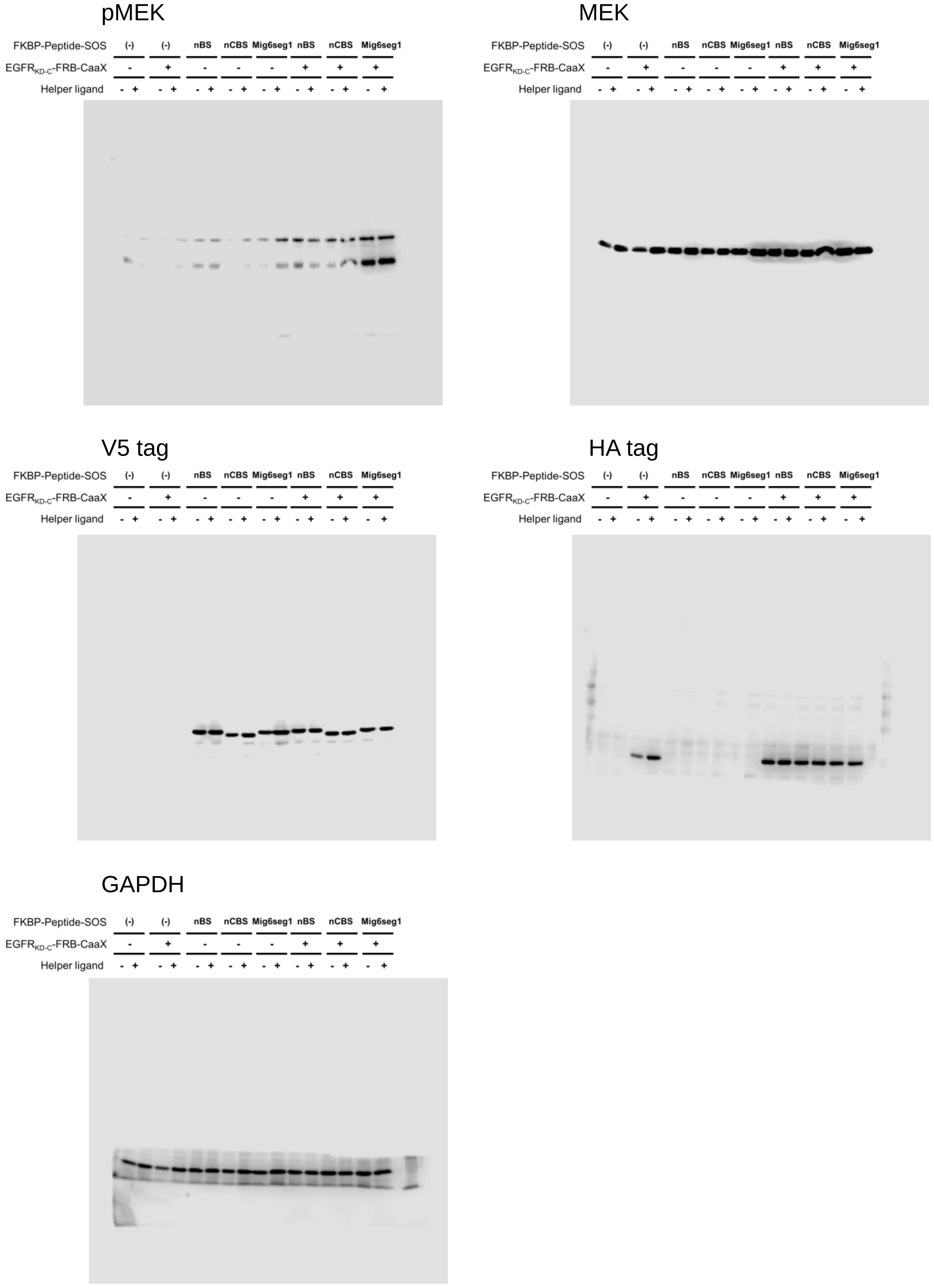
pMEK
MEK
V5 tag
HA tag
GAPDH
FKBP-Peptide-SOS
(-) (-) nBS nCBS Mig6seg1 nBS nCBS Mig6seg1
EGFR$_{KD-C}$-FRB-CaaX
- + - - - + + +
Helper ligand
- + - + - + - + - + - + - + - +

Fig. 5C

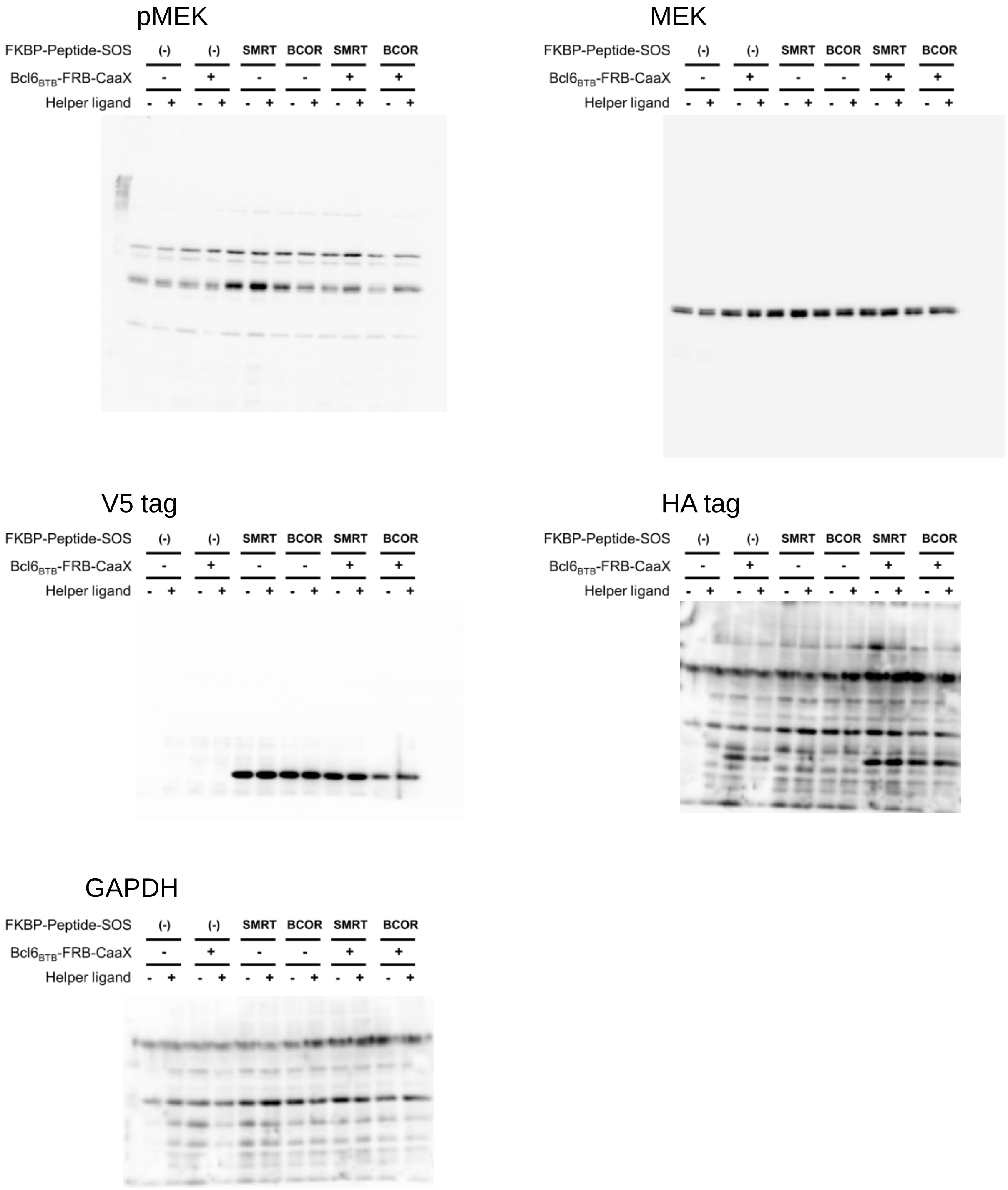

Supplementary Figure 3. Uncropped blot images.

The closely cropped original blot images are derived from membranes cut prior to hybridization with antibodies.
